# SARS-CoV-2 in Pregnancy—The First Wave

**DOI:** 10.3390/medicina57030241

**Published:** 2021-03-05

**Authors:** Andreia de Vasconcelos Gaspar, Isabel Santos Silva

**Affiliations:** Bissaya Barreto Maternity-Obstetrics Service B, Coimbra Hospital and University Center, 3000 Coimbra, Portugal; mariaisabelsantossilva@gmail.com

**Keywords:** SARS virus, coronavirus infections, pregnancy, pregnancy outcome, pregnancy complications, infectious

## Abstract

*Background and Objectives:* COVID-19, a disease caused by SARS-CoV-2, is a public health emergency. Data on the effect of the virus on pregnancy are limited. *Materials and Methods:* We carried out a retrospective descriptive study, in order to evaluate the obstetric results on pregnant women in which SARS-CoV-2 was detected through RT-PCR of the nasopharyngeal swab, at admission to the maternity hospital. *Results:* From 16 March to 31 July 2020, 12 SARS-CoV-2 positive pregnant women have been hospitalized. Eleven were hospitalized for initiation or induction of labor, corresponding to 0.64% of deliveries in the maternity hospital. One pregnant woman was hospitalized for threatened abortion, culminating in a stillbirth at 20 weeks of gestation. Regarding the severity of the disease, nine women were asymptomatic and three had mild illness (two had associated cough and one headache). Three had relevant environmental exposure and a history of contact with infected persons. None had severe or critical illness due to SARS-CoV-2. There were no maternal deaths. The following gestational complications were observed: one stillbirth, one preterm labor, one preterm prelabor rupture of membranes, and one fetal growth restriction. Four deliveries were eutocic, two vacuum-assisted deliveries and five were cesarean sections. The indications for cesarean section were obstetric. *Conclusions:* SARS-CoV-2 infection was found in a minority of hospitalized pregnant women in this sample. Most are asymptomatic or have mild illness, from gestational complications to highlight stillbirth and preterm birth. There were no cases of vertical transmission by coronavirus.

## 1. Introduction

Severe acute respiratory syndrome coronavirus 2 (SARS-CoV-2) is responsible for COVID-19 disease. Coronaviruses are RNA viruses. Sequencing of the complete genome and phylogenic analysis indicate that the coronavirus causing COVID-19 is a betacoronavirus, of the same subgenus as the severe acute respiratory syndrome (SARS) virus. It emerged in late 2019 in Wuhan and spread rapidly, resulting in a worldwide pandemic [1]. There are currently more than 20 million infected persons and 800,000 deaths. In Portugal, the first confirmed case of coronavirus infection occurred on 2 March 2020. Since then, there have been more than 600,000 cases in our country, with around 11,000 deaths occurring at the end of our data records. Transmission of the SARS-Cov-2 virus appears to occur from person to person, through respiratory droplets or contaminated surfaces. Some authors suggest that it can also be transmitted by air, through the inhalation of particles smaller than the droplets, which remain in the air [2,3]. Pregnant women appear to be particularly susceptible to respiratory infections due to the physiological changes that occur during pregnancy (elevation of the diaphragm, increased oxygen consumption, and edema of the respiratory tract mucosa), which make them particularly intolerant to hypoxia. This was seen in the influenza pandemic in 1918 and in the H1N1 pandemic in 2009, in which pregnant women had a higher rate of hospitalization and mortality than the general population [4,5]. In pregnancy, there is little data about COVID-19, the information being based mainly on case reports and small series. Portugal was one of the countries in Europe with the lowest number of COVID-19 and, consequently, data on this infection in pregnancy is limited.

This study aims to evaluate obstetric outcomes in women in whom SARS-CoV-2 was detected at admission to the maternity hospital, in the center region of the country.

## 2. Materials and Methods

This paper is a descriptive, retrospective study on pregnant women in which SARS-CoV-2 was detected on admission, from 16 March to 31 July 2020, in a Maternity of a Central Hospital in the Center Region of Portugal. The SARS-CoV-2 virus pandemic was enacted in mid-March 2020, and the hospital contingency plan in Coimbra started on 16 March 2020. Since then, testing has been carried out on pregnant women with symptoms compatible with COVID-19 (cough, fever ≥ 38 °C, headache, myalgia or dyspnea) or with high risk contacts, namely with infected cohabitant, or distancing less than 1 m and more than 15 min with a potentially infected person, according to the recommendations of ACOG (American College of Obstetricians and Gynecologists) and CDC (Centers for Disease Control and Prevention) [6,7]. On 10 April 2020, SARS-CoV-2 testing began for all pregnant women admitted to our maternity hospital. The test was carried out on the 1962 pregnant woman admitted to the maternity hospital, by searching for SARS-CoV-2 RNA by reverse transcription polymerase chain reaction (RT-PCR) on a nasopharyngeal swab specimen. It was performed previously (≤48 h) or at the time of hospital admission. The test was carried out by an obstetrician and analyzed by the laboratory of the clinical pathology service. The remaining clinical information was collected from electronic medical records. Newborns of mothers with SARS-CoV-2 underwent research of this infection by RT-PCR of a nasopharyngeal sample, carried out during delivery and 24 to 48 h afterwards.

Fetal death is considered that which occurs after 20 weeks gestation. There is no international consensus on the gestational age and birth weight necessary to report fetal deaths, so we opted for the definition of ACOG.

The primary outcome was to determine the severity of the condition and maternal mortality. Secondary outcomes consisted of determining the number of spontaneous abortions, preterm births, cesarean sections, and verifying whether vertical transmission occurred.

The constitution and manipulation of the database, as well as the statistical analysis, were performed using the SPSS program version 23 (IBM Corp., Armonk, NY, USA). A descriptive analysis of the distribution of patients was carried out, considering various sociodemographic variables. Categorical variables are presented as frequencies and percentages and continuous variables as means and standard deviations or medians and interquartile ranges. The test for normal data distribution was performed using the Shapiro–Wilk test or by analyzing the values of skewness and kurtosis.

## 3. Results

Since the beginning of the pandemic, 12 SARS-CoV-2 positive pregnant women have been admitted. Only one was diagnosed prior to the testing start, as she had symptoms compatible with COVID-19 (cough). Of all the pregnant women admitted to the maternity hospital, 0.61% were positive for SARS-CoV-2 (12/1962). Eleven were hospitalized for beginning or inducing labor, corresponding to 0.64% of the 1720 births that took place at the maternity hospital in this period. One pregnant woman was hospitalized at 20 weeks for threatened abortion, culminating in a stillbirth that same week. The median gestational age at diagnosis was 40 weeks (IQR 3), the majority being term pregnancies (≥37 weeks, *n* = 10, 83.3%). All pregnancies were unifetal. The demographic characterization is described in Table 1.

The mean maternal age was 36 years (SD 4.100). Most women were multiparous and healthy. None developed gestational diabetes or hypertensive disorders of pregnancy, namely pre-eclampsia and HELLP syndrome. Three had relevant environmental exposure and a history of contact with infected persons. Regarding the severity of the disease (National Institutes of Health), nine women were asymptomatic and three had a mild illness (two had associated cough and one headache). None had severe or critical illness due to SARS-CoV-2. The complementary diagnostic methods performed are shown in Table 2. 

An analytical study was carried out on seven pregnant women (58%), with lymphopenia in two cases and elevated liver parameters, AST (aspartate aminotransferase) and ALT (alanine aminotransferase), in only one. There was no need for admission to the Intensive Care Unit (ICU) or deaths due to COVID-19. The following gestational complications were observed: one stillbirth, one preterm labor and one preterm prelabor rupture of membranes (PPROM). The average gestational age of delivery was 40 weeks, with only one preterm birth. Preterm birth (PB) occurred after PPROM at 36 weeks. There were no cases of spontaneous PB before 34 weeks of gestation. Induction of labor was performed in three cases, two for 41 weeks and one due to prolonged rupture of membranes (>18 h). Four deliveries were eutocic, two vacuum-assisted deliveries and five were cesarean sections. The indications for cesarean section were obstetric: two for intrapartum fetal distress, one for failed labor induction, one for suspected fetopelvic incompatibility, and one due to being nulliparous with breech presentation. All women in labor received neuraxial anesthesia. As a postpartum complication, we highlight a uterine atony that was difficult to control, with consequent hypovolemic shock and the need for admission to the intensive care unit (ICU). This was the only woman who had a chest X-ray, which showed no abnormalities.

The average weight of newborns (NB) was 3121 g (SD 550.256), with an average APGAR index at the 5th minute of 9.73 (SD 0.467). There were no cases with an Apgar below 7 at the 5th minute. There were no congenital anomalies. All NB underwent SARS-CoV-2 research, with no case of vertical transmission. According to the guidance of Direção Geral da Saúde, the newborns stayed with their mothers, in asymptomatic and light cases, and the cradle was placed about 2 m from the mother’s bed. The mothers followed all the recommended isolation rules, namely the constant use of a mask and frequent hand washing.

At the time of discharge from the maternity hospital, all women were hemodynamically stable, with no need for oxygen supplementation or other therapy.

## 4. Discussion

The prevalence of infection in Portugal and, particularly, in our maternity hospital is lower than that reported in the literature (0.64%). In New York, about 15% of tested pregnant women were positive for SARS-CoV-2, and in Seattle and Washington the prevalence was around 3% [8,9]. In our sample, most pregnant women admitted to the maternity hospital were asymptomatic (75%). In other studies, the percentage of asymptomatic pregnant women admitted to the maternity hospital who tested positive for SARS-CoV-2 is variable, between 13 and 45% [2,10]. Data on small series and clinical cases suggest that pregnancy does not worsen the clinical course of COVID-19 [1,11]. 

Symptoms associated with COVID-19 are similar to the non-pregnant population: fever, cough, dyspnea, anosmia, ageusia, odynophagia, myalgia, headache, rhinorrhea or nasal congestion, nausea or vomiting, diarrhea, fatigue, confusion, and chest pain [12]. In this study, we had three symptomatic pregnant women: two with cough and one with headache. Of the pregnant women with COVID-19, all had a mild illness, according to the classification of the National Institutes of Health [13]. We have had no serious or critical cases requiring ICU admission due to COVID-19, contrary to what is reported by other countries. A systematic review indicates that the percentage of pregnant women with severe pathology is 15% and critical pathology 1.4%, with our prevalence being significantly lower, despite our small sample [14]. 

There were no deaths in our sample. The risk of death in pregnant women appears to be similar to the general population [8]. Most of these pregnant women were healthy and, since they had a mild illness, they did not need to be admitted to the Infectious Disease Service after delivery. Only pregnant women with moderate disease and comorbidities (hypertension, diabetes, chronic kidney disease, cardiopulmonary disease, immunosuppression) or severe illness require hospitalization [15]. ACOG recommends that a detailed clinical evaluation should be performed on pregnant women with respiratory impairment. Since in our population there were no pregnant women with respiratory impairment, complementary means of diagnosis were not performed in most situations.

Laboratory findings normally present include lymphopenia (47%) and a slight increase in liver enzymes (17%) [6]. The causes of these changes are unknown. Elevated liver markers may be associated with a virus-induced cytotoxic effect or immune-mediated inflammation [16]. In this study, with a small sample, we only found lymphopenia in two pregnant women and elevated liver enzymes in one case. These changes seem to revert spontaneously, without therapy [17]. However, the American Association for the Study of Liver Diseases (AASLD) states that all possible etiologies should be considered [18]. In the obstetric, overlap with other pathologies is, namely HELLP syndrome and pre-eclampsia [19]. The elevation of acute phase markers is also reported in the literature as a potential COVID-19 marker, namely PCR (C-reactive protein), vs. (erythrocyte sedimentation rate), lactate dehydrogenase (LDH), and ferritin [2]. However, there are no studies on these changes in pregnant women positive for SARS-CoV-2, and it is important to consider the physiological change of these markers in pregnancy, which are usually elevated [20]. 

Some authors suggest that chest radiography be performed for the initial evaluation of pulmonary complications in pregnant women hospitalized with COVID-19. However, CDC and ACR (American College of Radiology) do not recommend performing chest imaging for the diagnosis of COVID-19, but only in selected cases, due to their variable and non-specific findings [21]. In our institution, given the isolation measures of these women and the fact that the radiology service is in another hospital, no chest X-ray was performed in any case upon admission. The only one who underwent a chest X-ray was on admission to the ICU due to hypovolemic shock.

Regarding pregnancy complications, which refer to a stillbirth, a preterm birth, a PPROM, and a fetal growth restriction. The risk of preterm birth appears to be increased in pregnant women infected with SARS-CoV-2, occurring in about 20% of pregnancies [6]. Most preterm births are iatrogenic [22]. In this sample, preterm birth occurred at 36 weeks, after premature rupture of membranes in a nulliparous woman with a fetus in breech presentation, which is the reason why a cesarean section was performed. The development of fetal growth restriction is a theoretical concern and has been described with other SARS infections [23]. In COVID-19, there is little data available on fetal growth after maternal infection. Although significant placental histopathological changes are not universally present, suboptimal fetal growth is plausible because COVID-19 has been associated with poor uteroplacental vascular perfusion [24]. Although we have a stillbirth in this series, the abortion rate does not appear to be higher in infected pregnant women [25,26]. In this sample, delivery took place with an average GA of 40 weeks. In asymptomatic women or those with mild illness, the time of delivery should be determined only by obstetric indication. In this sample, we had four eutocic deliveries, two vacuum-assisted deliveries and five deliveries by cesarean section (45%). Our rate of cesarean sections is lower than that of the literature (50% to 85%).

Regarding uterine atony as a postpartum complication, it is not reported in the literature as having an association with SARS-CoV-2 infection [27]. Postpartum hemorrhage should be addressed according to the protocol in force at the institution.

Over 95% of newborns had no complications [9]. In this sample, there are no cases with an Apgar below 7 at the 5th minute, with an average Apgar of 9.73 (SD 0.467) at the 5th minute. Possible vertical transmission has been reported in several cases of maternal infection in the third trimester, suggesting that congenital infection is possible, but uncommon (<3% of maternal infections) [28]. Most neonatal infections are believed to result from respiratory droplets when neonates are exposed after delivery to mothers or other caregivers with SARS-CoV-2 infection. Congenital infection in the newborn was excluded by PCR of nasopharyngeal samples, which are performed at delivery and 24 to 48 h after delivery. There was no case of congenital infection.

However, recent studies have reported newborns with positive IgG and IgM antibodies, despite having a negative PCR, which suggests the possibility of vertical transmission [29,30]. More data is needed on this topic.

This study has the limitation of having a small sample of cases. In addition, most pregnant women were in the third trimester, having no information about the effect of the virus in the first and second semesters of pregnancy. The eventual histopathological analysis of the placenta could also add data about the mode of transmission of the virus. In our institution, this data was not available to contain the risk of contamination. Further studies are needed.

## 5. Conclusions

SARS-CoV-2 infection was found in a minority of hospitalized pregnant women. Most are asymptomatic or have a mild illness. 

From gestational complications, stillbirth and preterm birth are highlighted. There were no maternal deaths. There were no cases of vertical transmission by coronavirus.

Despite the apparently benign evolution of SARS-CoV-2 infection in this sample, until further studies are available, concern about COVID-19 in pregnancy should remain, since previous evidence with similar viruses has shown that the course can be severe and often deadly.

## Figures and Tables

**Table 1:** Demographic characterization.

Gestational Age at Diagnosis (weeks), *n* (%)		
	20	1 (8.3)
	36	1 (8.3)
	37	2 (16.7)
	39	1 (8.3)
	40	5 (41.7)
	41	2 (16.7)
Maternal age (years), mean (SD)		35.58 (4.100)
	Advanced maternal age (>35 years), *n* (%)	7 (58.3)
Nulliparity, *n* (%)		6 (50.0)
Gravida, mean (SD)		2.00 (1.279)
Parity, mean (SD)		0.75 (0.965)
Medical history, *n* (%)	depression	1
	hypothyroidism	1

**Table 2:** Complementary means of diagnosis.

Analytical Study, Mean (SD)		
	Leukocytes	12.03 (3.837)
	Lymphocytes	1.59 (0.818)
	C-reactive protein	3.16 (2.583)
	AST (aspartate aminotransferase)	28 (11.533)
	ALT (alanine aminotransferase)	16 (4.359)
Chest radiography, *n* (%)		1
